# Hypomagnesemia at the time of autologous stem cell transplantation for patients with diffuse large B-cell lymphoma is associated with an increased risk of failure

**DOI:** 10.1038/s41408-021-00452-0

**Published:** 2021-03-26

**Authors:** Jennifer J. Gile, Camden L. Lopez, Gordon J. Ruan, Matthew A. Hathcock, Jithma P. Abeykoon, Joy R. Heimgartner, Nikola A. Baumann, M. Molly McMahon, Ivana N. Micallef, Patrick B. Johnston, Jose C. Villasboas Bisneto, Luis F. Porrata, Jonas Paludo, Stephen M. Ansell, William J. Hogan, Thomas E. Witzig

**Affiliations:** 1 Division of Hematology, Department of Medicine, Mayo Clinic Rochester, Rochester, MN 55905 USA; 2 Department of Biomedical Statistics and Informatics, Mayo Clinic Rochester, Rochester, MN 55905 USA; 3 Division of Endocrinology, Department of Medicine, Mayo Clinic Rochester, Rochester, MN 55905 USA; 4 Department of Laboratory Medicine and Pathology, Mayo Clinic Rochester, Rochester, MN 55905 USA

**Keywords:** B-cell lymphoma, B-cell lymphoma

## Abstract

Magnesium is an essential element that is involved in critical metabolic pathways. A diet deficient in magnesium is associated with an increased risk of developing cancer. Few studies have reported whether a serum magnesium level below the reference range (RR) is associated with prognosis in patients with diffuse large B cell lymphoma (DLBCL). Using a retrospective approach in DLBCL patients undergoing autologous stem cell transplant (AHSCT), we evaluated the association of hypomagnesemia with survival. Totally, 581 patients eligible for AHSCT with a serum magnesium level during the immediate pre-transplant period were identified and 14.1% (82/581) had hypomagnesemia. Hypomagnesemia was associated with an inferior event-free (EFS) and overall survival (OS) compared to patients with a serum magnesium level within RR; median EFS: 3.9 years (95% CI: 1.63–8.98 years) versus 6.29 years (95% CI: 4.73–8.95 years) with HR 1.63 (95% CI: 1.09–2.43, *p* = 0.017) for EFS, and median OS: 7.3 years (95% CI: 2.91—upper limit not estimable) versus 9.7 years (95% CI: 6.92–12.3 years) with HR 1.90 (95% CI: 1.22–2.96, *p* = 0.005) for OS months 0–12, respectively. These findings suggest a potentially actionable prognostic factor for patients with DLBCL undergoing AHSCT.

## Introduction

Diffuse large B cell lymphoma (DLBCL) is the most common subtype of non-Hodgkin lymphoma (NHL)^1^. First-line treatment for DLBCL is typically based on rituximab plus cyclophosphamide, doxorubicin, vincristine, and prednisone (R-CHOP) backbone which cures approximately 60% of patients^2–4^. Patients who fail R-CHOP and meet the requirements for autologous hematopoietic stem cell transplant (AHSCT) are treated with salvage chemotherapy and if they have the sensitive disease, proceed to stem cell harvest and AHSCT^5–7^. AHSCT typically induces sustained remission in 40% of these patients; thus, relapse post-AHSCT remains an important issue^8^. The most important predictor of OS in these patients is the time from AHSCT to progression^9^. Efforts to improve the outcome of AHSCT have included changes in the stem cell collection technique to enrich immune cells^10–12^ and to enhance stem cell numbers^13^; the preparatory regimens have changed little. There have also been improvements in the therapy of those patients who relapse after AHSCT with the recent approval of CAR-T cell therapy^14^.

In our quest to discover other actionable prognostic factors in the AHSCT group, we turned to the evaluation of nutritional factors in light of the recent discovery of X-linked immunodeficiency with magnesium defect, Epstein–Barr virus, and neoplasia (XMEN) disease which suggests a role of magnesium in the development of hematologic malignancies and lymphocyte function^15^. XMEN is a primary immunodeficiency and congenital disorder of glycosylation^16^ caused by null mutations in the *MAGT1* gene which codes for a magnesium transporter that is a regulator of intracellular magnesium levels^17^. These studies demonstrate that magnesium deficiency leads to impaired immune reactivity^18^. Recently, we identified that hypomagnesemia at baseline is associated with an inferior OS in patients with Burkitt Lymphoma^19^. In order to evaluate the impact of hypomagnesemia in DLCBL outcome, we studied DLBCL patients proceeding to AHSCT since they typically have a pretransplant magnesium level, uniform conditioning, and specified standard post-AHSCT follow-up through year 1. Based on the data from XMEN disease and previous work that early absolute lymphocyte count (ALC) recovery predicts superior survival after AHSCT^10^, we hypothesized that patients with low magnesium levels prior to AHSCT would have a higher risk of relapse at day 100 and that hypomagnesemia would affect the recovery of lymphocytes (ALC recovery) at day +15.

## Methods

### Study population

The study was reviewed and approved by Mayo Clinic institutional review board. We utilized the Mayo Clinic Transplant Database to identify all patients greater than 18 years of age with a diagnosis of relapsed or refractory DLBCL that had failed first-line therapy, had the chemosensitive disease to salvage therapy, and were deemed eligible for AHSCT by the Lymphoma Transplant Physician group. The included patients underwent their first AHSCT in the Mayo Clinic Health Systems between January 1, 1998, and May 26, 2020. Prior to reinfusion of stem cells on day 0, all patients received a conditioning regimen which started on day −6. Using the database, we cross-referenced this search with patients who had a serum magnesium level (reference range: 1.7–2.6 mg/dL) performed in the Mayo Clinic clinical laboratory as part of their standard pretransplant evaluation workup between days −28 to −7. We chose days −28 to day −7 as it was typically the time period of stem cell evaluation and harvest while avoiding any effects of the conditioning chemotherapy on the magnesium level. Patients without serum magnesium in this window were excluded. Patients were considered to have hypomagnesemia if the magnesium level was less than the lower laboratory limit of 1.7 mg/dL. Electronic medical records were reviewed for demographics, baseline performance status, and the calcium, platelet count, creatinine, aspartate aminotransferase (AST), alkaline phosphatase, and ALC between 7 and 28 days prior to transplant. In the case of multiple values for these factors, the one closest to day −7 was used. These data were collected as part of the standard reporting guidelines of the center for international blood and marrow transplant research (CIBMTR) of which Mayo Clinic is a member.

### Statistical analysis

The association of hypomagnesemia with clinical and demographic factors was assessed using logistic regression. Variables with a *p* value < 0.1 in univariate analysis were included in a multivariate analysis. The association of hypomagnesemia and other factors with post-transplant survival was assessed using Cox proportional hazards regression. The primary survival outcome was overall survival (OS). OS was defined as the time from transplant until death or last follow-up. The secondary outcome was event-free survival (EFS) with events defined as relapse, re-transplantation, or death due to any cause. For the outcomes of 100-day and 1-year OS and EFS, event times were censored at 100 days and at 365 days, respectively, if an event (or loss of follow-up) had not occurred by that time. We used these relatively short follow-up times as we hypothesized that hypomagnesemia at the time of diagnosis would be associated with early clinical failure and also that long-term survival would be more difficult to link to a single pretransplant magnesium level. Survival curves were plotted with Kaplan–Meier methodology^20^. The relationship between magnesium level and OS or EFS was further investigated using Cox proportional hazards models in which continuous magnesium levels were modeled with a cubic spline with three knots to allow for nonlinearity. *p* Values were two-sided and were not adjusted for multiple testing. All statistical analyses were performed using R version 3.6.2.

## Results

### Patient characteristics

Of 739 patients with relapsed or refractory DLBCL undergoing AHSCT at Mayo Clinic during this time period, 581 (78.6%) patients had a magnesium level available prior to their conditioning regimen and were eligible for inclusion in the analyses. Of these patients, 14.1% (82/581) had hypomagnesemia with a median magnesium level of 1.6 mg/dL (1.0–1.6); the remaining 86% had magnesium levels within the RR with a median level of 1.9 mg/dL (1.7–2.6). The median number of days prior to undergoing transplant that a magnesium level was collected was 7 days (7–28 days).

A comparison of baseline characteristics of patients with hypomagnesemia vs. magnesium level within RR is included is summarized in Table 1. Patients were categorized according to the American Society of Blood and Marrow Transplantation risk categories for patients undergoing AHSCT. Totally, 87.7% of patients with low serum magnesium were considered low risk compared to 83.5% of patients with serum magnesium levels within RR (*p* = 0.41).

**Table 1:** Pretransplant laboratory values in all patients and by normal or low pretreatment serum magnesium level categories.

Variables	All patients	Mg within the reference range	Low Mg	*p* Value*	Multivariate analysis
	*N* = 581	(1.7–2.3 mg/dL)	(<1.7 mg/dL)		
		*N* = 499	*N* = 82		
Age, years; median (range)	62 (19–78)	62 (19–78)	62 (23–78)	0.09	0.38
*Gender (%)*					
Male	60.80%	62.50%	50%	0.03	0.015
Female	39.20%	37.50%	50%		
Race (% Caucasian)	91.20%	91.60%	89.00%	0.45	
Magnesium; median (range)	1.9	1.9	1.6		
	(1.0–2.6)	(1.7–2.6)	(1.0–1.6)		
% BEAM conditioning regimen	78.30%	77.20%	82.50%	0.33	
% Peripheral blood stem cell source	95.80%	96.30%	98.40%	0.22	
% Patients with calcium <8.5 or >10.0 mg/dL	41 (7.1%)	33 (7.5%)	8 (11.1%)	0.3	
Pretransplant ALC × 10(9)/L; median (range)	0.83 (0.11–11.94)	0.85 (0.11–11.94)	0.81 (0.15–5.23)	0.77	
% Patients with ALC <0.5 × 10^9^/L pretransplant	139 (23.9%)	120 (24.1%)	19 (23.2%)	0.85	
Platelet count × 10^9^/L; median (range)	119.0 (10.0–669.0)	122.5 (10.0–669.0)	95.0 (15.0–315.0)	<0.001	<0.001
% Patients with platelet count <150 × 10^9^/L	400 (68.6%)	336 (68.9%)	64 (80%)	0.045	
% Patients with Albumin ≤3.5 g/dL	19 (3.3%)	16 (4.1%)	3 (4.7%)	0.81	
% Patients with AST >50 U/L	48 (8.3%)	43 (8.6%)	5 (6.1%)	0.44	
% Patients with ALP ≥130 U/L	403 (69.4%)	342 (68.7%)	61 (74.4%)	0.3	
Creatinine mg/dL; median (range)	0.9 (0.4–2.9)	0.9 (0.4–2.5)	0.95 (0.40–2.90)	0.09	0.03
% Patients with creatinine ≥ 1.3 mg/dL	66 (11.4%)	52 (10.4%)	14 (17.1%)	0.08	

*p Value between normal and low magnesium groups.

Abbreviations: *ALC* absolute lymphocyte count, *AST* aspartate aminotransferase, *ALP* alkaline phosphatase, *Mg* magnesium, BEAM (carmustine (BCNU), etoposide, cytosine arabinoside, and melphalan).

On univariate analysis, male gender (OR 0.599, 95% CI: 0.374–0.959; *p* = 0.033) and higher platelet count (OR 0.518 per doubling, 95% CI: 0.376–0.706; *p* < 0.001) were significantly associated with having serum magnesium levels within RR; higher ALP (OR 1.464 per doubling, 95% CI: 1.029–2.103; *p* = 0.036) was associated with a low serum magnesium level. Totally, 122 patients were missing a corresponding serum albumin level within the day −28 to day −7 timeframe; thus, the multivariate analyses were performed with and without serum albumin levels. Multivariate logistic regression on baseline variables (excluding albumin) with *p* < 0.1 on univariate regression demonstrated that gender (*p* = 0.011), platelets (*p* < 0.001), and creatinine (*p* = 0.020) were significantly associated with magnesium levels (Table 1). When albumin was included, multivariate logistic regression on baseline variables with *p* < 0.1 on univariate regression demonstrated that only platelets (*p* < 0.001) were significantly associated with magnesium level.

### Overall and event-free survival

Median follow-up following transplantation was 3.0 years (range: 0.1–21.3 years). Since the focus was on the role of a single pretransplant magnesium level on EFS and OS, we evaluated the magnesium level with respect to two key landmarks used in AHSCT—day +100 and 1 year.

**Fig. 1:**
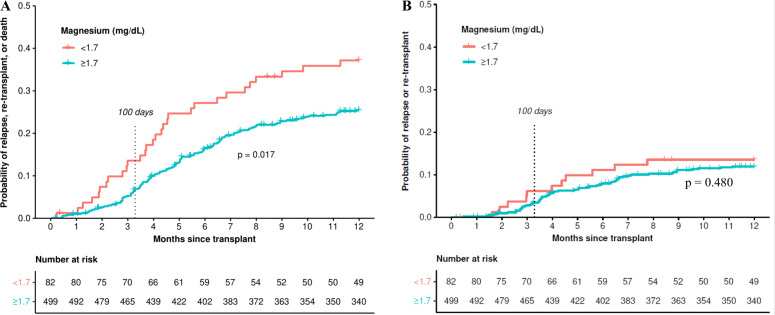
Event free and relapse free survival curves. **A** Event-free survival of patients with diffuse large B-cell lymphoma undergoing autologous stem cell transplant by premyeloablative serum magnesium level between 1998 and 2020. Log-rank test *p* = 0.017. **B** Relapse free survival of patients with diffuse large B-cell lymphoma undergoing autologous stem cell transplant by premyeloablative serum magnesium level between 1998 and 2020. Log-rank test *p* = 0.480.

For the outcome of EFS (Fig. 1A), 7.8% (45/581) of patients had an event by day 100 and 26.3% (153/581) had an event by month 12. Of these 153 patients with an event during the 12 months following SCT, 55% (84/153) died without known relapse or retransplant; an additional 29 (19%) deaths were associated with relapse; and 40 (26%) relapses/retransplants did not die in the first year. Median EFS for patients with hypomagnesemia was estimated to be 3.9 years (95% CI: 1.63–8.98 years) vs. 6.29 years (95% CI: 4.73–8.95 years) for patients with serum magnesium levels within RR. Day +100 EFS was estimated to be 86.4% (95% CI: 79.3–94.2%) in those patients with hypomagnesemia vs. 93.1% (95% CI: 90.9–95.4%) in patients with serum magnesium levels within RR. EFS at 1 year for patients with hypomagnesemia was estimated at 62.8% (95% CI: 53.1–74.3%) compared to 74.6% (95% CI: 70.8–78.6%) for patients with serum magnesium levels within RR. For EFS up to 1 year, the hazard ratio (HR) for hypomagnesemia estimated from a Cox model was 1.63 (95% CI: 1.09–2.43, *p* = 0.017). The impact of hypomagnesemia on EFS was primarily on non-relapse mortality (Fig. 1B); there was no significant difference in lymphoma relapse rate between patients with hypomagnesemia and serum magnesium levels within the RR (*p* = 0.480).

**Fig. 2:**
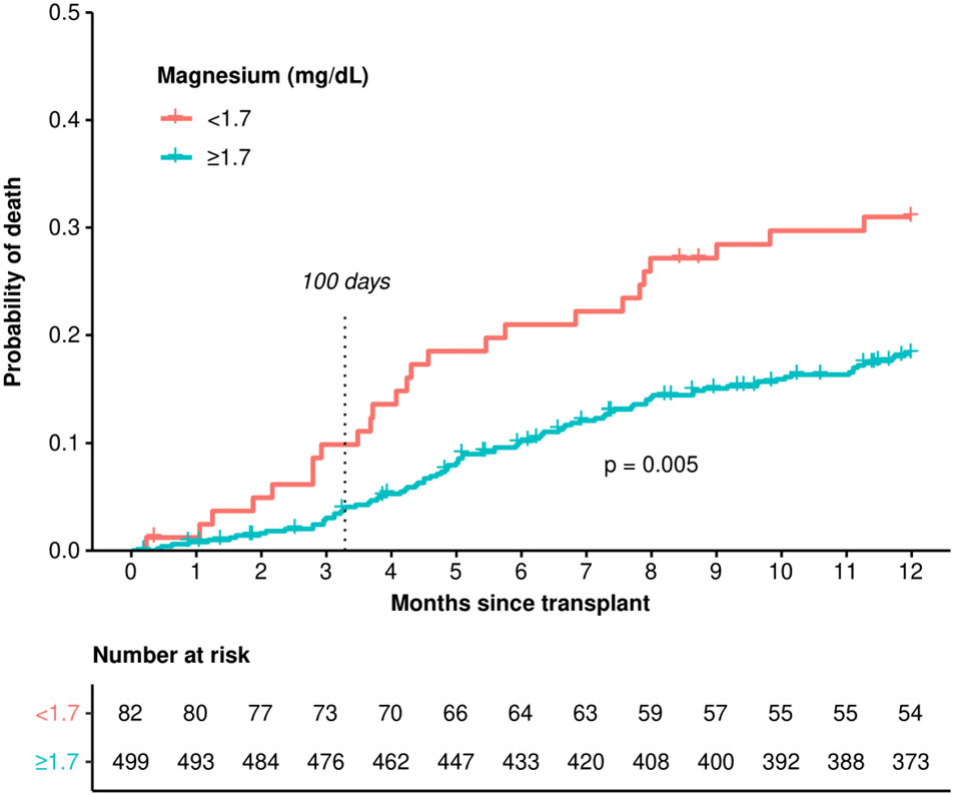
Overall survival of patients with diffuse large B-cell lymphoma undergoing autologous stem cell transplant by premyeloablative serum magnesium level between 1998 and 2020. Log-rank test *p* = 0.005.

There were 28 deaths (4.8%) observed in the first 100 days following transplantation, and 113 (19.4%) within the first year. OS at day 100 and 12 months was also worse for those with baseline hypomagnesemia. The median OS was estimated to be 7.3 years (95% CI: 2.91—upper limit not estimable) for patients with hypomagnesemia vs. 9.7 years (95% CI: 6.92–12.3 years) for patients with serum magnesium levels within RR. Day 100 OS for patients with hypomagnesemia vs. those with serum magnesium levels within RR was estimated at 90.1% (95% CI: 83.9–96.9%) and 96.0% (95% CI: 94.2–97.7%), respectively. OS at 1 year for patients with hypomagnesemia vs. serum magnesium levels within RR was 69.0% (95% CI: 59.6–79.9%) and 81.7% (95% CI: 78.3–85.3%), respectively. For OS up to 1 year, the estimated HR for hypomagnesemia was 1.90 (95% CI: 1.22–2.96, *p* = 0.005) (Fig. 2).

**Fig. 3:**
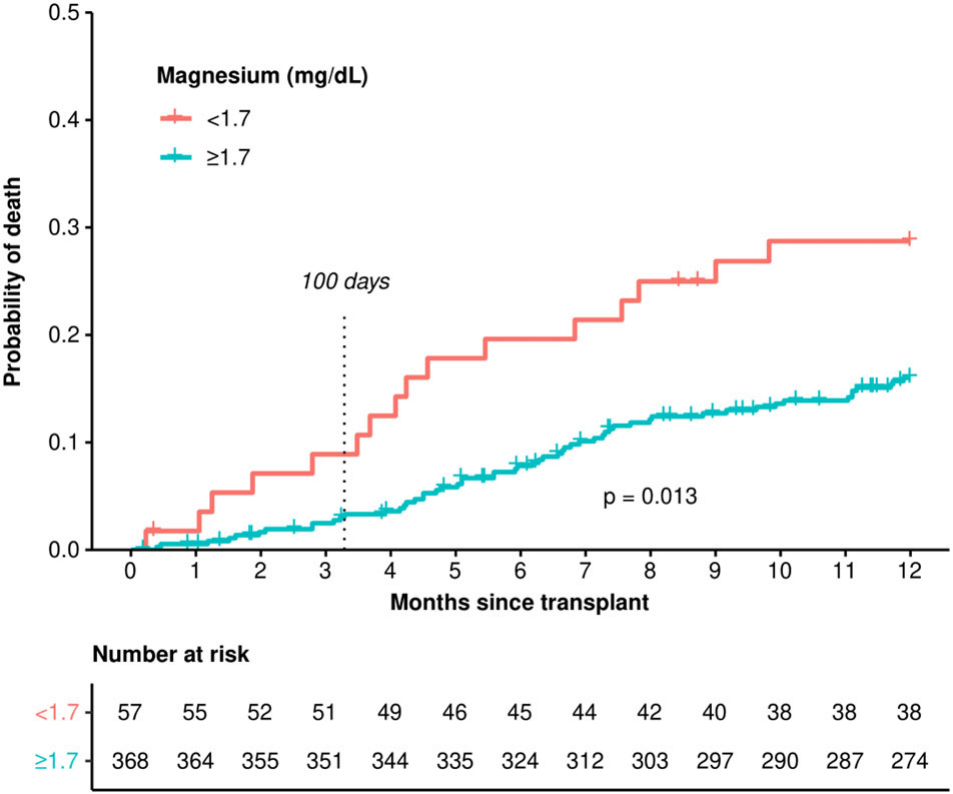
Overall survival of patients with diffuse large B-cell lymphoma undergoing autologous stem cell transplant by premyeloablative serum magnesium level between 2010 and 2020. Log-rank test *p* = 0.013.

Our cohort spans a 22-year time period and some standard of care transplant practices have changed in the last 2 decades. We, therefore, performed a survival analysis restricted to patients transplanted in the last 10 years. For patients with hypomagnesemia treated between 2010 and 2020 (*n* = 425), the OS HR for hypomagnesemia was 2.03 (95% CI: 1.16–3.54), *p* = 0.013 (Fig. 3). For patients treated 1998–2010 (*n* = 156), the OS HR for hypomagnesemia trended in a similar direction at 1.60 (95% CI: 0.77–3.36), *p* = 0.2.

Table 2 shows the univariate and multivariate analyses of factors related to OS up to 1-year post AHSCT. Higher serum magnesium (HR 0.199 per doubling, 95% CI: 0.078–0.503; *p* < 0.001) and higher albumin (HR 0.090 per doubling, 95% CI: 0.021–0.394; *p* = 0.001) were associated with lower mortality rates; whereas, higher serum creatinine (HR 1.669 per doubling, 95% CI: 1.066–2.614; *p* = 0.025) was associated with greater mortality up to 1-year post-AHSCT. In a multivariate Cox regression without albumin, lower magnesium (*p* < 0.001) and higher creatinine (*p* = 0.038) were independent predictors of inferior OS (Table 2). Calcium levels and liver function tests were not significantly associated with OS. When albumin was included, both lower magnesium (*p* = 0.004) and lower albumin (*p* = 0.002) were independent predictors of inferior OS.

**Table 2:** Univariate and multivariate analysis of vs overall survival at 1 year (Cox proportional hazards regression model using continuous variables).

	Univariate analysis			Multivariate analysis		
Variables	HR	95% CI	*p* Value	HR	95% CI	*p* Value
Age, per 10 years	0.99	0.85–1.15	0.89			
Male gender	1.26	0.85–1.85	0.25			
Higher serum magnesium values	0.2	0.08–0.50	<0.001	0.21	0.08–0.53	<0.001
Higher absolute lymphocyte count at baseline	0.9	0.77–1.06	0.22			
Higher platelet count at baseline	0.84	0.66–1.06	0.15			
Higher serum albumin	0.09	0.02–0.39	0.001			
Higher serum creatinine	1.67	1.07–2.61	0.03	1.61	1.03–2.52	0.04

**Fig. 4:**
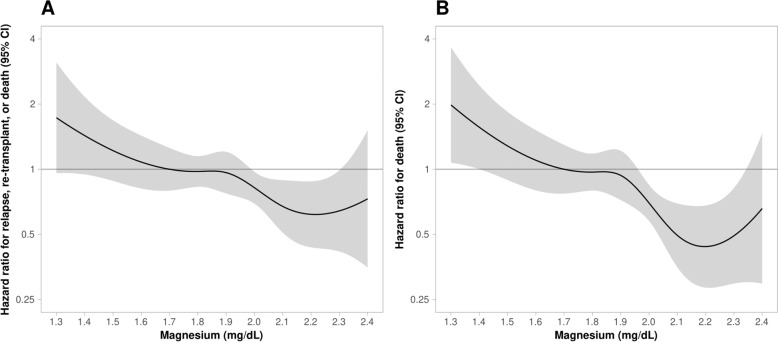
Hazard of death estimated as a function of magnesium level. Log-scale hazard of **A** event-free survival and **B** overall survival up to 1 year following transplant by serum magnesium (mg/dL) drawn prior to myeloablative conditioning. The shaded areas represent the confidence intervals.

Since a low serum magnesium level predicted an inferior outcome after AHSCT, we investigated what level of serum magnesium was best for predicting low vs. high-risk patients. Using proportional hazards regression of EFS and OS (Fig. 4) on the continuous magnesium level and using cubic splines to allow for a nonlinear relationship, we determined that a magnesium level of 2.0 mg/dL should be considered as a reasonable target for future studies. The hazard of death (or relapse or retransplantation), estimated as a function of magnesium level, decreased most rapidly as magnesium increased through 2.0 mg/dL (Fig. 4).

**Fig. 5:**
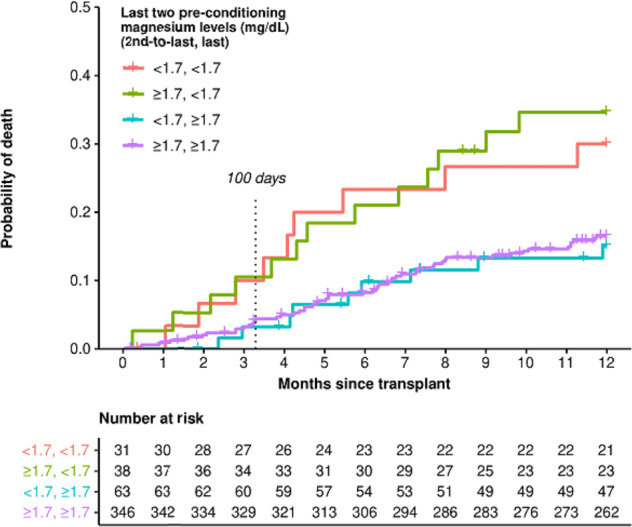
Overall survival stratified by magnesium levels. Overall survival of patients with diffuse large B-cell lymphoma undergoing autologous stem cell transplant by the two most recent premyeloablative serum magnesium levels.

Finally, we performed analysis restricted to patients with at least two magnesium levels prior to undergoing conditioning in preparation for AHSCT to see whether the combination or trend of multiple magnesium levels might be more informative about survival than the last magnesium level alone. As demonstrated in Fig. 5, the value closest to day −7 was most predictive of outcome.

### Day +15 ALC recovery after transplant

There was no significant difference between baseline ALC values in patients with serum magnesium levels within RR vs. low magnesium levels (Table 1). In addition, the day +15 ALC recovery level between patients with serum magnesium levels within RR and low serum magnesium levels was not significantly different (Table 3).

**Table 3:** Day 15 lymphocyte recovery by premyeloablative serum magnesium level.

Day 15 absolute lymphocyte recovery	Mg within the reference range	Low Mg	*p* Value
	*N* = 499	*N* = 82	
Absolute lymphocyte count < 0.5×10^9^/L	221 (44.4%)	39 (47.6%)	0.591
Absolute lymphocyte count > 0.5 × 10^9^/L	277 (55.6%)	43 (52.4%)	

*Mg* magnesium.

## Discussion

Abnormalities of serum magnesium are common in hospitalized patients. A very large study found that of over 11,000 patients hospitalized with malignant diseases, 33% had hypomagnesemia and those patients had a higher risk of in-hospital mortality^21^. Serum magnesium, although commonly included in laboratory studies in hospitalized patients, is not routinely measured in new, untreated lymphoma patients in the outpatient setting. However, serum magnesium is measured in the AHSCT setting and replaced as needed during the peritransplant period. The goal is to normalize the serum magnesium with no specific target other than to achieve a value within the RR. The increase in-hospital mortality with hypomagnesemia in addition to the association of XMEN disease with lymphoma led to this study in DLBCL patients receiving AHSCT. Indeed, we found that 14% of patients with DLBCL undergoing AHSCT had hypomagnesemia pretransplant and they had inferior outcomes at day 100 and at 1 year. The exact cause of the hypomagnesemia is difficult to discern in this patient population but is likely due to poor dietary intake due to symptoms from relapsed DLBCL, diuretics, renal dysfunction, and previous platinum-based chemotherapy^22,23^. We also found that over half of the events that the patients experienced in the first year were not associated, as best we could discern, with relapse of the DLBCL. This indicates a potential role for magnesium in all-cause mortality post-DLBCL SCT as shown by others for all hospitalized patients^21^. This finding is similar to studies on the gut microbiome in patients undergoing allogeneic stem cell transplant where the microbial diversity changes impacted primarily non-relapse mortality and not a relapse of the tumor^24^.

There have been only a few other studies of magnesium and lymphoma but none specifically in DLBCL or the transplant setting. Merza et al.^25^ studied 55 patients with blood cancers (7 NHL) and found 4/7 to have a low level compared to control patients. In addition, our group also recently investigated the incidence of hypomagnesemia in 61 patients with Burkitt Lymphoma prior to chemotherapeutic treatment and found hypomagnesemia in 16%. Those patients with hypomagnesemia also had an inferior OS^19^.

Our study also found a significant association between low serum magnesium levels and thrombocytopenia. Others have shown thrombocytopenia to be a predictor for poor prognosis in patients with DLBCL^26^; however, thrombocytopenia in our dataset was not predictive of the inferior OS at 1 year. We are not aware of a mechanism that would link magnesium to megakaryocyte function thus this association likely reflects the disease state and prior treatment the patient received. Elevated creatinine was also associated with low magnesium levels and both increased creatinine and lower magnesium were independently associated with inferior OS. These associations are understandable given the kidney’s role in magnesium homeostasis via reabsorption at the Loop of Henle^27^. Renal handling of magnesium is affected when kidney function declines^23^.

Serum albumin is also a prognostic marker in DLBCL^28^. Alterations in circulating albumin levels have been shown to affect magnesium levels^29^, similar to calcium as about one-third of extracellular magnesium is bound to proteins^18^. However, we did not find an association between hypoalbuminemia and hypomagnesemia in this study.

Based on previous studies examining the role of magnesium and the immune system, we hypothesized that hypomagnesemia patients with DLBCL undergoing ASCHT would have poor outcomes and that this could be associated with impaired lymphocyte recovery after stem cell reinfusion. Previous studies have found that the day 15 ALC (ALC-15) after AHSCT is a significant predictor for survival in DLBCL^10^. From a numerical standpoint, we did not find any difference in ALC recovery between the magnesium sufficient and deficient groups. However, magnesium is an important nutrient for immune function^30^ and it is possible that lymphocyte function (not absolute number) could be impaired due to low serum magnesium leading to impaired EFS/OS. Future studies will need to include functional studies along with magnesium replacement to better understand these associations and mechanism(s).

The strengths of our study include a large cohort at one institution with the same lymphoma transplant team evaluating each patient for selection for AHSCT. As a result, there was a unified approach to patient selection and treatment. In addition, the median follow-up time after transplantation was 3.0 years with a focus on year 1. We selected early endpoints to compare with serum magnesium pretransplant as patients who fail SCT typically do so early.

There are some limitations to our study. The patients were accrued over a 22-year period; however, the conditioning regimens have changed little over that time period and we demonstrated that our findings are still relevant in the most recent decade (Fig. 3). We also were unable in this retrospective study to clearly determine the cause of the magnesium deficiency. Although platinum-based chemotherapy can impair renal function, the impact of low serum magnesium on outcome was demonstrated to be independent of albumin and creatinine. We also did not evaluate the role of magnesium levels determined after the start of the conditioning regimen nor after day 0 stem cell reinfusion. After these time points, the patients were evaluated and treated daily by the team, and magnesium deficiency typically replaced with IV or oral replacement.

These data provide a rationale to consider a magnesium replacement strategy for hypomagnesemia patients going to transplant. If a replacement is commenced, what should be the target? Our study provides valuable and useful data for clinicians. The hazard of death for these DLBCL patients, estimated as a function of magnesium level, decreased most rapidly as the serum magnesium increased to 2.0 mg/dL. Although 1.7 mg/dL is technically within the reference range, in a subset of cardiovascular patients, levels less than 2.0 mg/dL have been shown to be associated with an increased risk of sudden cardiac death^31,32^. In general, cardiology patients have magnesium levels aggressively replaced to a target of 2.0 mg/dL, because of this increased risk of cardiac death^33^. In this DLBCL AHSCT study, the hazard rate for relapse reached 1 at the 1.9–2.0 mg/dL level indicating that a level of 2.0 mg/dL is also a reasonable target. Recent work has also suggested that the current reference range of magnesium is too low^34^ and further research into the conventional serum magnesium levels is needed.

Although we have demonstrated the importance of low magnesium with respect to outcome, the key next question is whether magnesium replacement in this subset (14%) of relapsed DLBCL patients with hypomagnesemia will actually improve outcome. A low magnesium level may simply be another biomarker of illness and replacement of magnesium to the reference range levels prior to AHSCT may not improve outcomes. Interestingly, we found that the magnesium level just prior to conditioning therapy and AHSCT is most important for predicting outcomes in patients after transplant, regardless of previous serum magnesium levels. Thus, magnesium replacement at this important time point needs to be addressed prospectively given that this is a low-cost approach to further improve AHSCT outcomes. The peri-transplant period offers an ideal time to test these future magnesium strategies as diet and magnesium levels can be carefully monitored in the transplant setting. Lastly, we were unable to demonstrate the impact of hypomagnesemia on ALC recovery at day 15. Future studies should evaluate magnesium levels in association with T- and B-cell function as it is likely that it is the functional defect that is more important than the numerical deficiency.
